# Isoliquiritigenin suppresses human melanoma growth by targeting miR-301b/LRIG1 signaling

**DOI:** 10.1186/s13046-018-0844-x

**Published:** 2018-08-06

**Authors:** Shijian Xiang, Huoji Chen, Xiaojun Luo, Baichao An, Wenfeng Wu, Siwei Cao, Shifa Ruan, Zhuxian Wang, Lidong Weng, Hongxia Zhu, Qiang Liu

**Affiliations:** 1 School of Traditional Chinese Medicine, Southern Medical University, Guangzhou, 510515 China; 2 Integrated Hospital of Traditional Chinese Medicine, Southern Medical University, Guangzhou, 510315 China

**Keywords:** Isoliquiritigenin, Melanoma, miR-301b, LRIG1, Apoptosis

## Abstract

**Background:**

Isoliquiritigenin (ISL), a natural flavonoid isolated from the root of licorice (*Glycyrrhiza uralensis*), has shown various pharmacological properties including anti-oxidant, anti-inflammatory and anti-cancer activities. MicroRNAs (miRNAs), a class of small non-coding RNAs, have been reported as post-transcriptional regulators with altered expression levels in melanoma. This study aims to investigate the anti-melanoma effect of ISL and its potential mechanism.

**Methods:**

We investigated the effect of ISL on the proliferation and apoptosis of melanoma cell lines with functional assays, such as CCK-8 assay, colony formation assay and flow cytometry. The protein level of apoptosis related genes were measured by western blotting. High-throughput genome sequencing was used for screening differentially expressed miRNAs of melanoma cell lines after the treatment of ISL. We performed functional assays to determine the oncogenic role of miR-301b, the most differentially expressed miRNA, and its target gene leucine rich repeats and immunoglobulin like domains 1 (LRIG1), confirmed by bioinformatic analysis, luciferase reporter assay, western blotting and immunohistochemical assay in melanoma. Immunocompromised mouse models were used to determine the role of miR-301b and its target gene in melanoma tumorigenesis in vivo. The relationship between miR-301b and LRIG1 was further verified in GEO data set and tissue specimens.

**Results:**

Functional assays indicated that ISL exerted significant growth inhibition and apoptosis induction on melanoma cells. MiR-301b is the most differentially expressed miRNA after the treatment of ISL and significantly downregulated. The suppressive effect of ISL on cell growth is reversed by ectopic expression of miR-301b. Intratumorally administration of miR-301b angomir enhances the inhibitory effect of ISL on tumor growth in vivo. Bioinformatic analysis showed that miR-301b may target LRIG1, miR-301b suppresses the luciferase activity of reporter constructs containing 3’UTR of LRIG1 as well as the expression level of LRIG1. And the anti-cancer effect of ISL is mitigated when LRIG1 is silenced in vivo and in vitro. Analysis of the melanoma samples obtained from patients shows that LRIG1 is negatively correlated with miR-301b.

**Conclusions:**

ISL may inhibit the proliferation of melanoma cells by suppressing miR-301b and inducing its target LRIG1.

**Electronic supplementary material:**

The online version of this article (10.1186/s13046-018-0844-x) contains supplementary material, which is available to authorized users.

## Background

Melanoma is the most aggressive type of skin cancer that originates from melanocytes, accounting for a high mortality rate annually [1]. It is well known that surgical resection is the most common treatment for melanoma, but it is mostly invalid for patients with advanced melanoma, and the prognosis is poor with a 5-year survival rate < 16% [2]. For the treatment of advanced malignant melanoma, comprehensive and multidisciplinary approach should be applicable, such as chemotherapy, radiotherapy and immunotherapy [3, 4]. But little benefit was obtained from these regimens mainly because of the lack of effective biomarkers [5]. It has attracted increasing attention that looking for new biomarkers and exploring the potential molecular mechanism of melanoma development is pivotal for the treatment of malignant melanoma.

MicroRNAs (miRNAs) are a class of small non-coding RNAs that play an important role in posttranscriptional gene regulation by cleaving or repressing the translation of their target mRNAs [6]. Accumulating evidence has corroborated that miRNAs are involved in many physiological and pathological processes including cell proliferation, invasion, migration, apoptosis, differentiation, and metabolism [7, 8]. Some studies have verified that miRNAs are involved in the development and progression of different types of tumors, either as agonist or antagonist [9–11]. Extensive research of functions and the mechanism of miRNAs in melanoma has been carried out in recent years. Ectopic expression of miR-211 in melanoma cell lines has been shown to impose the inhibitory effect on growth, invasion and metastasis of melanocytes [9–12]. Overexpression of miR-196a was shown to reduce the invasive capacity of melanoma cells [13]. MiR-18b and miR-149 induced the cell apoptosis of melanoma by targeting p53. Reduced expression of miR-210 was indicated to facilitate the escape of melanoma cells from cytotoxic T lymphocytes [12, 14]. MiR-let-7a, a tumor suppresser, decreased the cellular proliferation by reducing the expression of cyclin-dependent kinases [15]. MiR-30b, miR-30d, miR-145 and miR-182 were found to regulate cell invasion and metastasis in melanoma [16–18]. Some researchers have demonstrated that miRNAs are involved in epigenetic modification. Methylation of miR-375, miR-34b and miR-182 was shown to be tightly correlated with high stage melanoma, leading to enhance cell invasiveness and motility [12, 19, 20].

Isoliquiritigenin (ISL), a natural flavonoid isolated from the root of licorice (Glycyrrhiza uralensis), has a chalcone structure (4, 20, 40-trihydroxychalcone) [21]. ISL has lots of biological properties, such as anti-inflammatory, anti-oxidant, anti-platelet aggregation, vasorelaxant, and estrogenic effects [22, 23]. Some researchers have found that ISL is an effective mitosis inhibitor and apoptosis inducer [24, 25]. Studies of the anti-cancer activity of ISL have been conducted for decades, being reported in ovarian cancer, prostate cancer, breast cancer, oral squamous cell carcinoma and colon cancer [26–29]. ISL has been shown to induce the reprogramming of human melanoma cells and inhibits the proliferation of mouse melanoma cells [30–32], but the anti-cancer effect of ISL on human melanoma remains elusive.

In this study, we showed that ISL significantly inhibited the growth and proliferation of melanoma cells, and miR-301b is predicted to be the most relevant miRNA. Next, we confirmed that miR-301b attenuated the anti-cancer effect of ISL on melanoma in vivo and in vitro by functionally targeting LRIG1. Intratumorally silence of LRIG1 mitigated the apoptosis induced by ISL. Finally, we notarized that LRIG1 and miR-301b are negatively correlated in human melanoma progression. These findings provide a proof of concept that ISL exerts anti-proliferation and pro-apoptosis effect on melanoma by suppressing miR-301b and inducing the target LRIG1.

## Methods

### Cell culture and tissue specimens

Human melanoma cell line(A375, A2058) obtained from American Type Culture Collection (Manassas, VA, US) was maintained in Dulbecco’s modified Eagle’s medium containing 10% fetal bovine serum and cultured at 37 °C in 5% CO_2_. Primary human melanomas and normal skin specimens were obtained from patients who were diagnosed in Integrated Hospital of Traditional Chinese Medicine, Southern Medical University. All patients who provided melanomas and normal skin specimens provided full consent for the study. Each cancer specimen contained at least 80% tumor cells, as confirmed by microscopic examination. Tissues were preserved by snap-freeze and stored at − 80 °C for subsequent protein and RNA extraction for western blotting and RT-qPCR analysis as per instructions. This study was approved by the ethics committee of the Southern Medical University.

### Cell proliferation assay

CCK-8 assay was performed to evaluate the viability and proliferation of melanoma cells after Isoliquiritigenin (ISL) treatment. To evaluate the viability of melanoma cells  after Isoliquiritigenin (ISL) treatment, A375/A2058 cells were inoculated in 96-well plate and treated with indicated concentration of ISL for 24 h, then the supernatant was removed and complete media containing 10% CCK-8 was added to each well, the 96-well plate was incubated at 37 °C for 2 h, the optical density was read by Varioskan LUX Multimode Microplate Reader (Thermofisher, USA). For the detection of cell proliferation of melanoma cells, A375/A2058 cells were treated with ISL for 24, 48 and 72 h, then the supernatant was removed and complete media containing 10% CCK-8 was added, the plate was incubated at 37 °C for 2 h and read the optical density.

### Plate colony formation assay

For colony formation assays, 600 cells were inoculated into 6-well plates with 2 mL Dulbecco’s modified Eagle’s medium supplemented with 10% FBS. After 14 days, the resulting colonies were rinsed with PBS, and then fixed with 4% paraformaldehyde for 10 min, and stained with Giemsa (Sigma, USA) for 40 min, then rinsed with PBS again. Only the visible colonies (diameter > 50 mm) were counted.

### Flow cytometry

Cell apoptosis was evaluated by flow cytometry(BD Biosciences, USA), A375 cells and A2058 cells were treated with indicated concentration of ISL for 24 h, respectively. 195 μL Annexin V-fluorescein isothiocyanate (Beyotime, China) and 5 μL propidium iodide were added according to the manufacturer’s protocol, and samples were then incubated for 10 min in dark place at room temperature prior to flow cytometric analysis (BD Accuri C6; software version 1.0.264.21; BD Biosciences).

### High-throughput genome sequencing

Total RNA was extracted with RNeasy Mini Kit (Cat# 74106, Qiagen) according to the instruction. An Agilent Bioanalyzer and a NanoDrop ND-2000 spectrophotometer 2100 (Agilent Technologies, Santa Clara, CA, USA) were used to determine the RNA quality. Total RNA(initial volume 1 μL) was used to establish a MiRNA library using the TruSeq Small RNA Sample Prep kit (Illumina, Inc). Sample preparation was performed using the acceptable miRNA library according to the method described in the Illumina HiSeq 2500 User Guide and the ultimate concentration for each sample was 10 pM.

### Lentiviral transfection

Lentivirus containing pre-miRNA-301b expressed in a lentiviral vector (pLKO.1-puro) were generated in 293 T cells as previously described [33]. Briefly, the coding oligo-nucleotides of antisense human miR-301b mimics and NC sequence were cloned and inserted into a lentivirus expression vector, pLKO.1-puro. The miR-301b mimics and NC viral particles were produced in 293 T cells (CRL-3216; ATCC, Manassas, VA, USA) via lentivirus expression vector co-expressed with pPACK packaging system (Systems Biosciences, Palo Alto, CA, USA). A375 and A2058 cells were transfected by miRNA 301b-GFP-expressing lentiviruses with the multiplicity of infection or MOI equals to 40. Transfection monitoring was performed by observing the GFP positive cells under a Fluorescent microscopy.

### In vivo tumor model

8-week-old immunocompromised mice were injected with 3 × 10^6^ A2058 cells into the flanks. The growth of the tumors was observed by naked eyes. After the tumor reached 80 mm^3^ in volume, the mice were divided into 3 treatment group at random: PBS + miR-301b angomir control (5 nmoL), ISL(20 mg/kg) + miR-301b angomir control (5 nmoL), ISL(20 mg/kg) + miR-301b angomir (5 nmoL). For the treatment of Si-LRIG1, mice were divided into 3 treatment group: PBS + si-NC, ISL + si-NC, ISL + si-LRIG1. MiR-301b/angomir and Si-LRIG1/NC was injected into the tumors while ISL into abdominal cavity every other day after the tumors reached the desired size. The animals were sacrificed after 6 weeks treatment and the tumors were excised for pathological examination.

### Histological analysis

Samples were fixed in 4% paraformaldehyde and then embedded in paraffin. Hematoxylin and eosin (H&E) staining was performed as previously described [34]. For immunohistochemistry analysis, the paraffin sections were deparaffinized, and cooked in citrate buffer (2.1 M citric acid, pH 6.0) at 120 °C for 30 min for antigen retrieval followed by incubation in 5% BSA(Sigma Aldrich, USA) to block nonspecific binding. And the sections were incubated in primary antibody overnight at 4 °C. The sections were then washed with PBS and incubated for 15 min at room temperature in a solution of anti-rabbit IgG (Abcam, UK). For detection of apoptosis index, sections were stained with TdT-mediated dUTP Nick-End Labeling(KeyGen BioTECH, China) according to the manufacturer’ instructions. Nucleus were stained with DAPI and the sections were processed using Histostain Plus and DAB kits, and the pictures were captured using a light microscope.

### Prediction of target genes of miR-301b

Melanoma-associated miRNA Microarray dataset GSE46517 and GSE15605 was downloaded from Gene Expression Omnibus (GEO) database (http://www.ncbi.nlm.nih.gov/geo/). The dataset GSE46517 and GSE15605, based on the platform as GPL96 and GPL570, respectively. GSE46517 included thirty-one tumor samples from melanoma patients and eight normal skin samples without melanoma as control, GSE15605 included forty-six tumor samples from melanoma patients and sixteen normal skin samples without melanoma as control. Differentially expressed genes (DEGs) were screened using the online tool GEO2R/R package limma, and DEGs between melanoma group and control group were screened and selected by the cut-off point of *P* < 0.05 and the [Log FC(fold change)] ≥ 1.5. TargetScan (http://www.targetscan.org) was used to predict the target genes of miR-301b. The common target genes predicted by TargetScan and GEO database were selected as potential target genes of miR-301b.

### Luciferase reporter assay

MiR-301b binding sites of LRIG1 3’UTRs were amplified by PCR from human total cDNA. The PCR product was then subcloned into NheI and SalI sites of pMIR-Report vector to generate the pmiR-LRIG1-Luc reporter constructs as described previously [35, 36]. Wild-type and mutagenic binding sequence of the target gene LRIG1 are listed in Additional file 1: Figure S2B. Constructed wild-type luciferase reporter (WT), mutant luciferase reporter(MUT) or empty vectors were co-transfected with miR-301b mimics or its corresponding negative control into MSC. The luciferase activity was assessed with a Double-Luciferase Reporter Assay Kit, purchased from Promega Biotech Co., Ltd. (Beijing, China), using the Dual-Light Chemiluminescent Reporter Gene Assay System (Berthold, Germany), which was normalized to firefly luciferase activity.

### RT-qPCR

Total RNA extraction was performed using the Trizol Reagent (Invitrogen, USA) according to the manufacturer’s instruction. The cDNAs were synthesized using a PrimeScript RT reagent kit (TaKaRa, Japan) with the following conditions: 37 °C for 15 min, 85 °C for 5 s, hold at 4 °C. For validation of the differentially expressed miRNAs, miRNA was reverse-transcribed using specific RT primers provided with the TaqMan MicroRNA Assay (Applied Biosystems). The reverse transcription was performed at following conditions: 16 °C for 30 min; 42 °C for 30 min, and 85 °C for 5 min. The miRNA cDNAs were amplified using the TaqMan Universal PCR master mix II (Applied Biosystems) with specific probe provided in the TaqMan Small RNA Assay (Applied Biosystems). This process was performed using ABI Prism 7500 HT sequence detection system (Applied Biosystems, Foster City, CA) at the conditions: 3 min at 95 °C, 15 s at 95 °C and 30 s at 60 °C for 40 cycles. The β-actin and U6 were used as loading controls for quantitation of mRNA and miRNAs, respectively. Sequences of mRNA primers used for RT-qPCR in this study were listed in Additional file 2: Table S1.

### Western blot

Proteins were extracted from cultured A375 and A2058 cells using RIPA solution (Beyotime, China) with protease inhibitor and phosphatase inhibitor. And protein concentration was measured using BCA Protein Assay Kit (Thermofisher, USA). Next, protein sample was separated via SDS-PAGE and electro-transferred onto PVDF membranes. Following blockade with 5% BSA, membranes were incubated with the following antibodies (all purchased from Abcam, UK) overnight at 4 °C:bcl-2 (1:1000), bax (1:1000), cleaved-parp (1:1000), cleaved-caspase-3 (1:1000),LRIG1 (1:1000), β-actin (1:1000). The membrane was then incubated with HRP-conjugated IgG (1:5000) for 2 h at room temperature. Immunoblots were quantified using Image Lab (version 2.0) software.

### Statistical analysis

Data with normal distribution were presented as mean ± SD. Student’s t-test was applied for comparison between two groups, and One-way ANOVA followed by Tukey comparison test was used for comparison between at least three groups. All statistical analyses were performed using SPSS 21.0 software. *P* < 0.05 was considered statistically significant.

## Results

### The effect of Isoliquiritigenin on the proliferation and apoptosis of melanoma cells in vitro

**Fig. 1:**
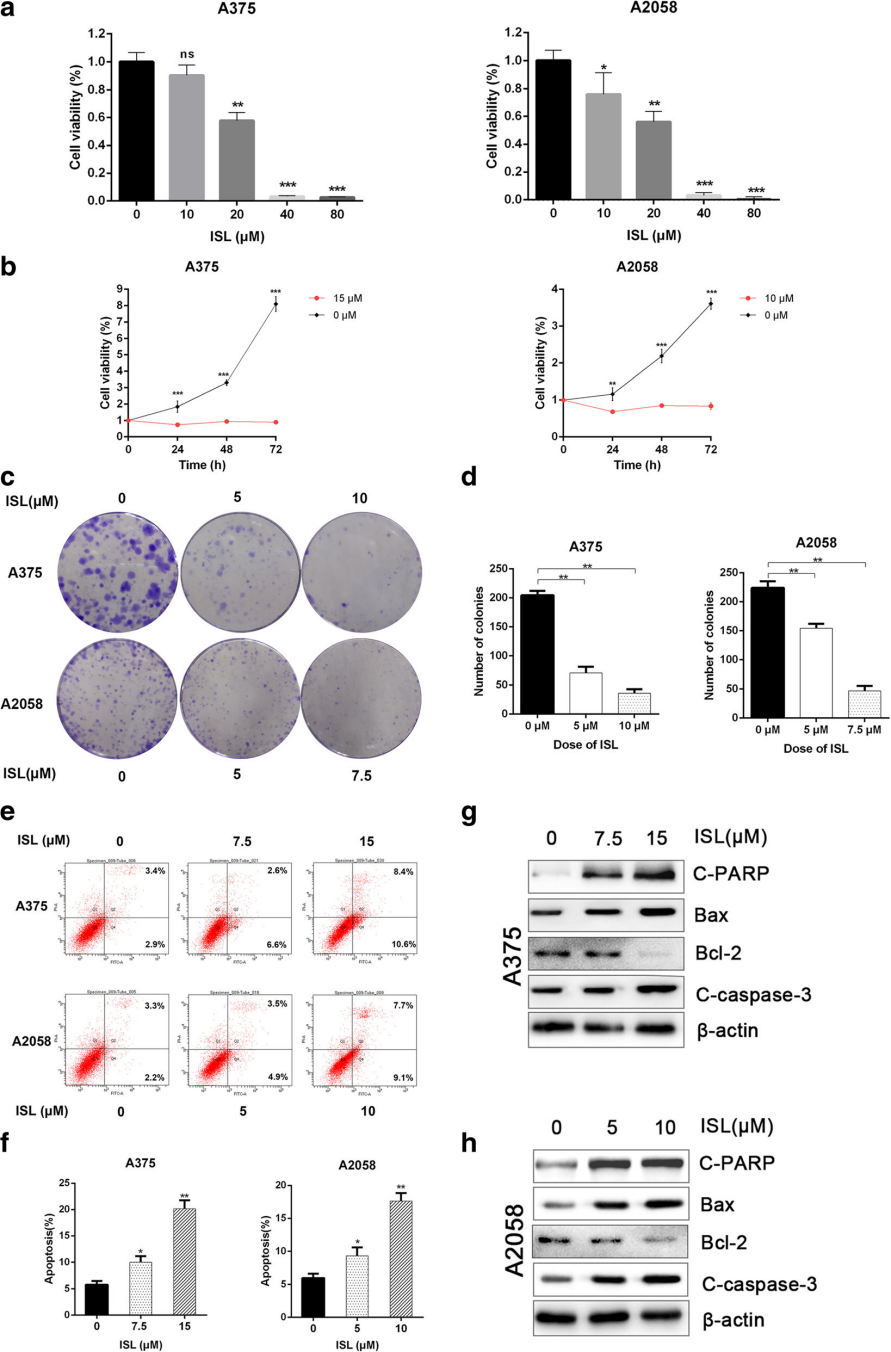
ISL inhibits cell proliferation and induces cell apoptosis in melanoma cells in vitro. **a** A375/A2058 cells were treated with ISL (0, 10, 20, 40, 80 μM) for 24 h, and cell viability was analyzed by CCK-8 assay. **b** A375 and A2058 cells were treated with 15 μM and 10 μM ISL respectively, cell proliferation at indicated time (24, 48, 72 h) was measured by CCK-8 assay. **c**, **d** Representative images and quantification of colony formation of A375 and A2058 cells after being treated with ISL (0, 5, 10 μM) and ISL (0, 5, 7.5 μM) respectively. **e**, **f** Flow cytometry analysis of apoptosis of A375 and A2058 cells after being treated with ISL (0, 7.5, 15 μM) and ISL (0, 5, 10 μM) for 24 h respectively. **g**, **h** Expression of apoptosis associated proteins (c-PARP, bcl-2, bax, C-caspase-3) of A375 and A2058 cells after being treated with ISL (0, 7.5, 15 μM) and ISL (0, 5, 10 μM) for 24 h respectively. *P < 0.05, **P < 0.01, ***P < 0.001. *n* = 3

To investigate the effect of ISL on the growth and apoptosis of melanoma cells, we treated A375 and A2058 cells with ISL of different concentration (0, 10, 20, 40, 80 μM) for 24 h, and the cell viability was detected using CCK-8 assay. As shown in Fig. 1a, ISL exhibited dose-dependent inhibitory on the viability of both cell lines. Furthermore, we treated A375 and A2058 cells with 15 μM and 10 μM ISL respectively for 24, 48 and 72 h, we found that ISL dramatically suppressed the proliferation of both cell lines (Fig. 1b). Next, we attempted to determine the impact of ISL on the colony forming capacity of two cell lines, the representative images and quantification of colonies are shown in Fig. 1c and d. In addition to its inhibitory effect on cancer cell proliferation, ISL also induced the apoptosis of both cell lines by increasing the expression of pro-apoptotic genes C-PARP, Bax, cleaved-caspase-3 (C-caspase-3) and decreasing the expression of anti-apoptotic gene Bcl-2 as shown in Fig. 1e, f, g, h and Additional file 3: Figure S1G, H, suggesting that ISL remarkably inhibited the proliferation and induced the apoptosis of melanoma cells in vitro. What’s more, we found that ISL remarkably suppressed the proliferation of MEWO, a cell line of BRAF wild type melanoma(Additional file 3: Figure S1A and B). ISL induced the apoptosis of MEWO, and also dramatically suppressed the colony forming capacity of MEWO (Additional file 3: Figure S1C, D, E and F).

### MiRNA expression profiles of melanoma cells treated with ISL

**Fig. 2:**
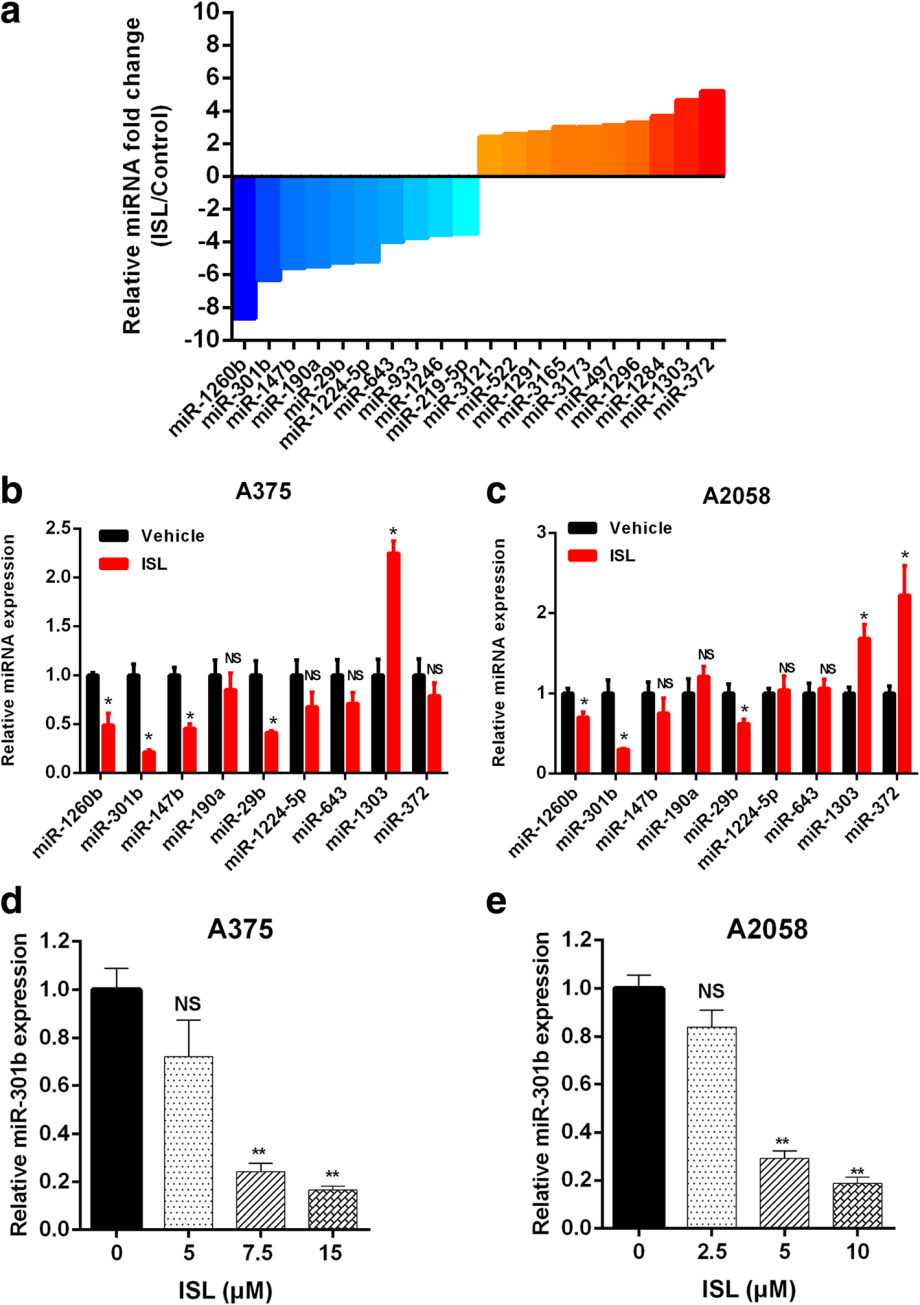
High-throughput sequencing analysis of differentially expressed miRNAs in A375 cell line treated with 7.5 μM ISL. **a** Top 10 upregulated and downregulated miRNAs in the ISL treated group compared with control group. **b**, **c** RT-qPCR analysis of the expression levels of miRNAs with fold change > 4. **d**, **e** RT-qPCR analysis of the expression level of miR-301b in ISL(0, 7.5, 15 μM) treated A375 cells and ISL(0, 5, 10 μM) treated A2058 cells, respectively. The data represented the mean ± SEM of three independent experiments. **P < 0.01 vs control; *P < 0.05 vs control; NS., not significant

High-throughput sequencing analysis was performed to identify the differentially expressed microRNAs treated with ISL, the top 10 upregulated and downregulated microRNAs were depicted in Fig. 2a. Next, we performed RT-qPCR to examine the expression level of top 7 downregulated and top 2 upregulated microRNAs in A375 and A2058 cells treated with ISL. MiR-301b exhibited the most significant expression change in the treatment of ISL(Fig. 2b, c). Furthermore, we examined the expression level of miR-301b in A375 and A2058 cells treated with ISL(0, 5, 7.5, 15 μM) and ISL(0, 2.5, 5, 10 μM) respectively, it showed that the expression of miR-301b decreased with a dose dependent manner(Fig. 2d, e). These results revealed that miR-301b was the most differentially expressed miRNA and its expression was dramatically inhibited by ISL.

### The effect of miR-301b on tumor growth in vitro

**Fig. 3:**
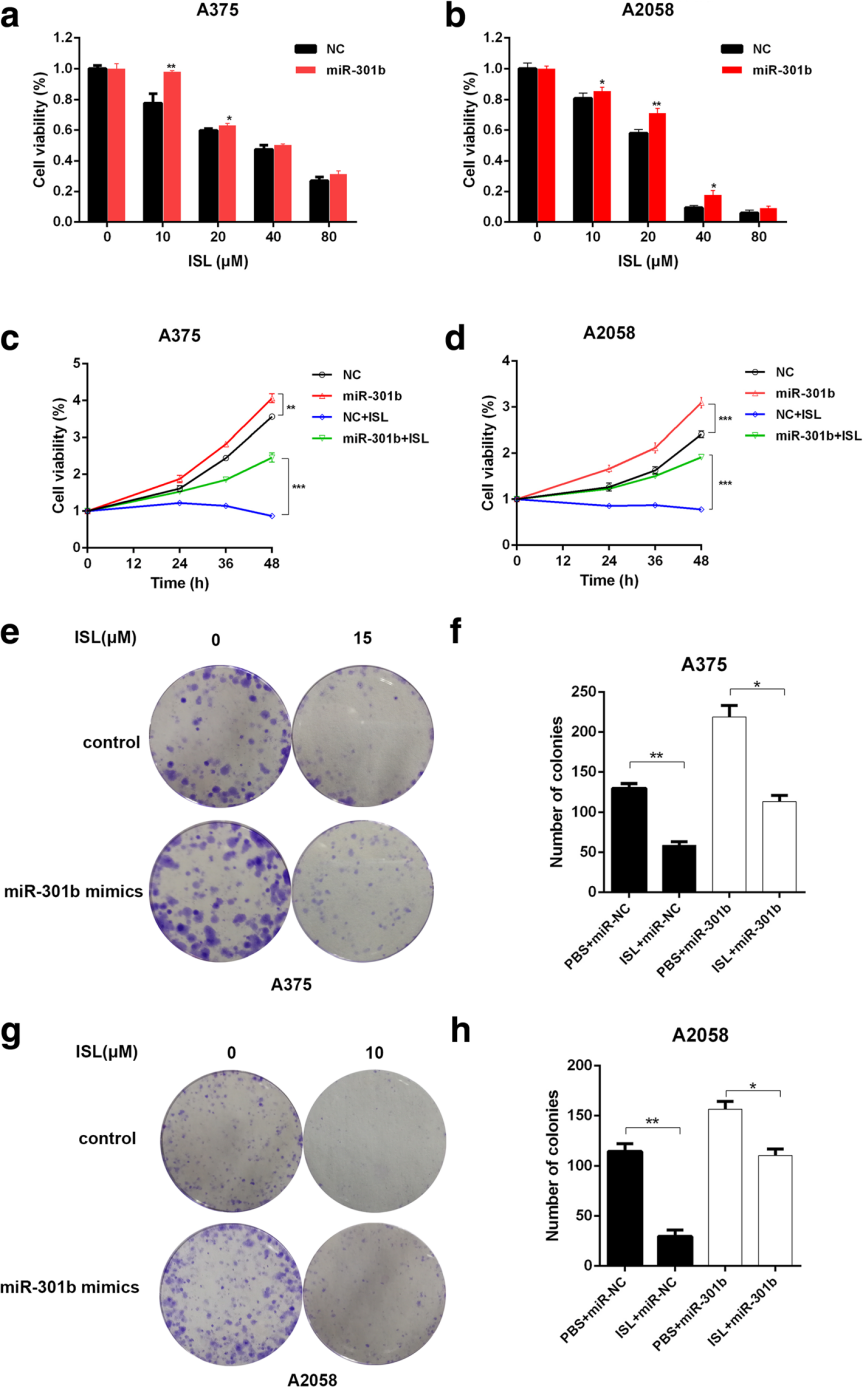
MiR-301b reverses the inhibition of ISL in melanoma. A375 and A2058 cells were transfected with miR-301b or control (NC). **a**, **b** A375/A2058 cells were treated with ISL (0, 10, 20, 40, 80 μM) for 24 h, and cell viability was analyzed by CCK-8 assay. **c**, **d** A375 and A2058 cells were treated with 15 μM and 10 μM ISL respectively, cell proliferation at indicated time (24, 48, 72 h) was measured by CCK-8 assay. **e**-**h** Representative images and quantification of colony formation of A375 and A2058 cells after being treated with ISL (0, 15 μM) and ISL (0, 10 μM) respectively. *P < 0.05, **P < 0.01, ***P < 0.001, *n* = 3

To investigate the effect of miR-301b on the proliferation and apoptosis of melanoma cells, we transfected A375 and A2058 cells with miR-301b-GFP-expressing lentiviruses and the established stable cell lines were used for the following experiment. RT-qPCR showed that ISL downregulated the expression of miR-301b in both wild type and miR-301b overexpressing melanoma cells (Additional file 1: Figure S2A, B). CCK-8 assay results indicated that the concentration-dependent inhibitory effect of ISL on melanoma cell viability and proliferation were attenuated by miR-301b (Fig. 3a, b, c, d). As shown in previous study, ISL exerted significantly suppressive effect on the colony forming capacity of melanoma cells, the phenomenon was alleviated by the ectopic expression of miR-301b(Fig. 3e, f, g, h). All the results above suggested that the anti-cancer activity of ISL against melanoma was probably mediated by miR-301b and the effect was weakened by the presence of miR-301b.

### The effect of miR-301b on melanoma cell apoptosis in vitro

**Fig. 4:**
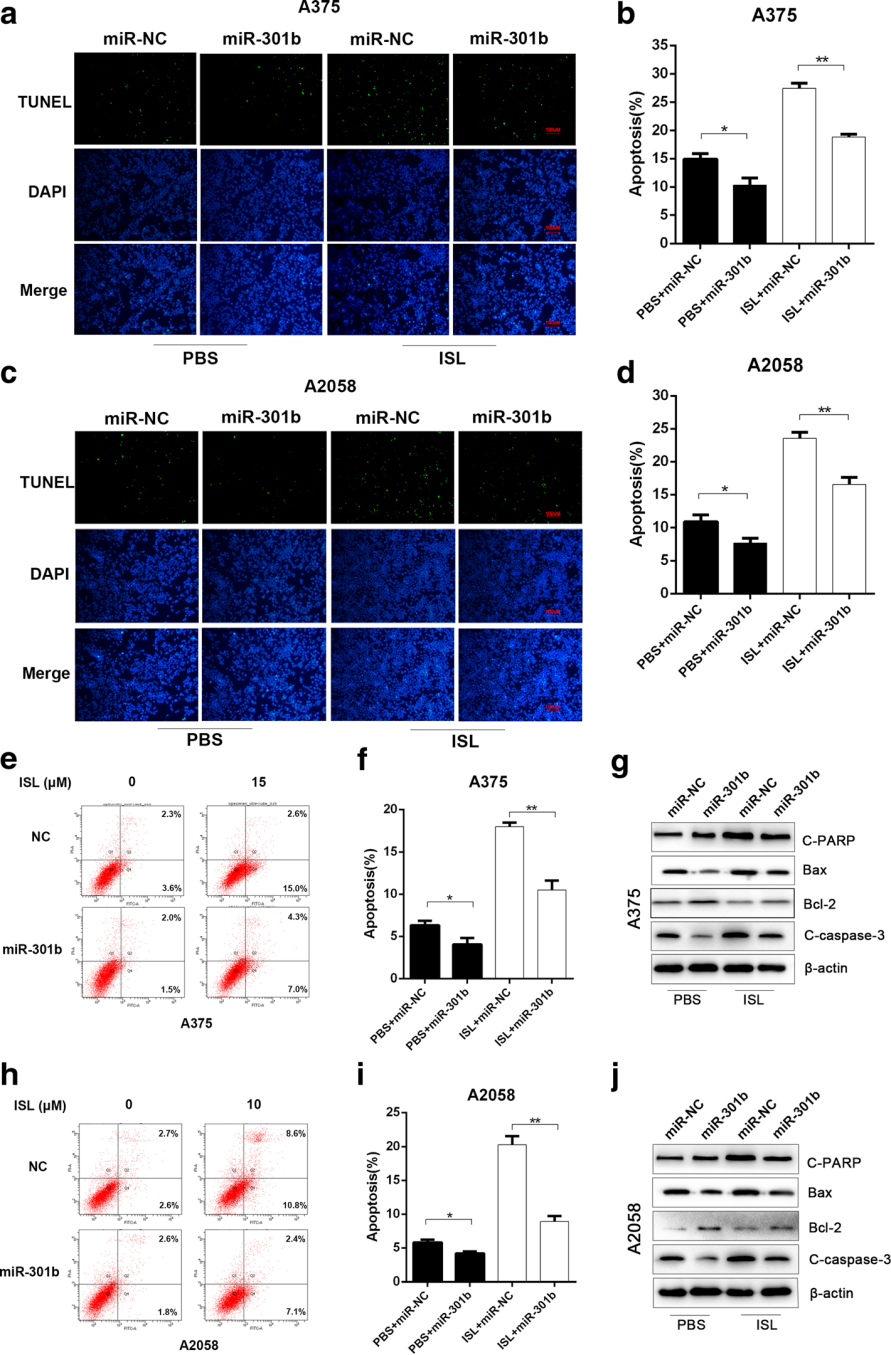
MiR-301b mitigated the ISL induced tumor cell apoptosis. A375 and A2058 cells were transfected with miR-301b or control(NC) (**a**-**d**) Representative images and quantification of TUNEL staining of A375 and A2058 cells after being treated with ISL (0,15 μM) and ISL (0,10 μM) respectively. **e**-**h** Flow cytometry analysis of apoptosis of A375 and A2058 cells after being treated with ISL (0, 15 μM) and ISL (0, 10 μM) for 24 h respectively. **i**, **j** Expression of apoptosis associated proteins (c-PARP, Bax, bcl-2, C-caspase-3) of A375 and A2058 cells after being treated with ISL (0, 15 μM) and ISL(0, 10 μM) for 24 h respectively. *P < 0.05, **P < 0.01, *n* = 3

To evaluate the effect of miR-301b on tumor cell apoptosis, we treated wild type or miR-301b-overexpressing A375/A2058 cells with either ISL or PBS treatment and performed TUNEL staining. As shown in Fig. 4a-d, ISL treatment significantly decreased the percentage of TUNEL-positive cells compared with PBS treatment. However, when tumor cells overexpressed miR-301b, the ISL induced apoptosis was remarkably mitigated in comparison with miR-NC group. Meanwhile, we performed flow cytometry to measure the apoptosis ratio of each group, we found the ISL induced cell death was reversed by miR-301b to a large extent, which was consistent with the TUNEL staining result (Fig. 4e-h). To further elucidate the underlying mechanism, we performed western blot to analysis the protein expression level of pro-apoptotic genes(C-PARP, Bax, C-caspase-3) and anti-apoptotic gene(Bcl-2) in both cell lines, the results demonstrated that ISL significantly increased the protein level of C-PARP, Bax, C-caspase-3 and decreased the protein level of Bcl-2, and the effect was notably reversed by miR-301b (Fig. 4i, j and Additional file 1: Figure S2C, D). These results collectively indicated that the pro-apoptotic effect of ISL on melanoma cells was prominently stored by the presence of miR-301b, and the inhibition of miR-301b may enhance the therapeutic effect of ISL on melanoma.

### The effect of miR-301b on tumor growth in vivo

**Fig. 5:**
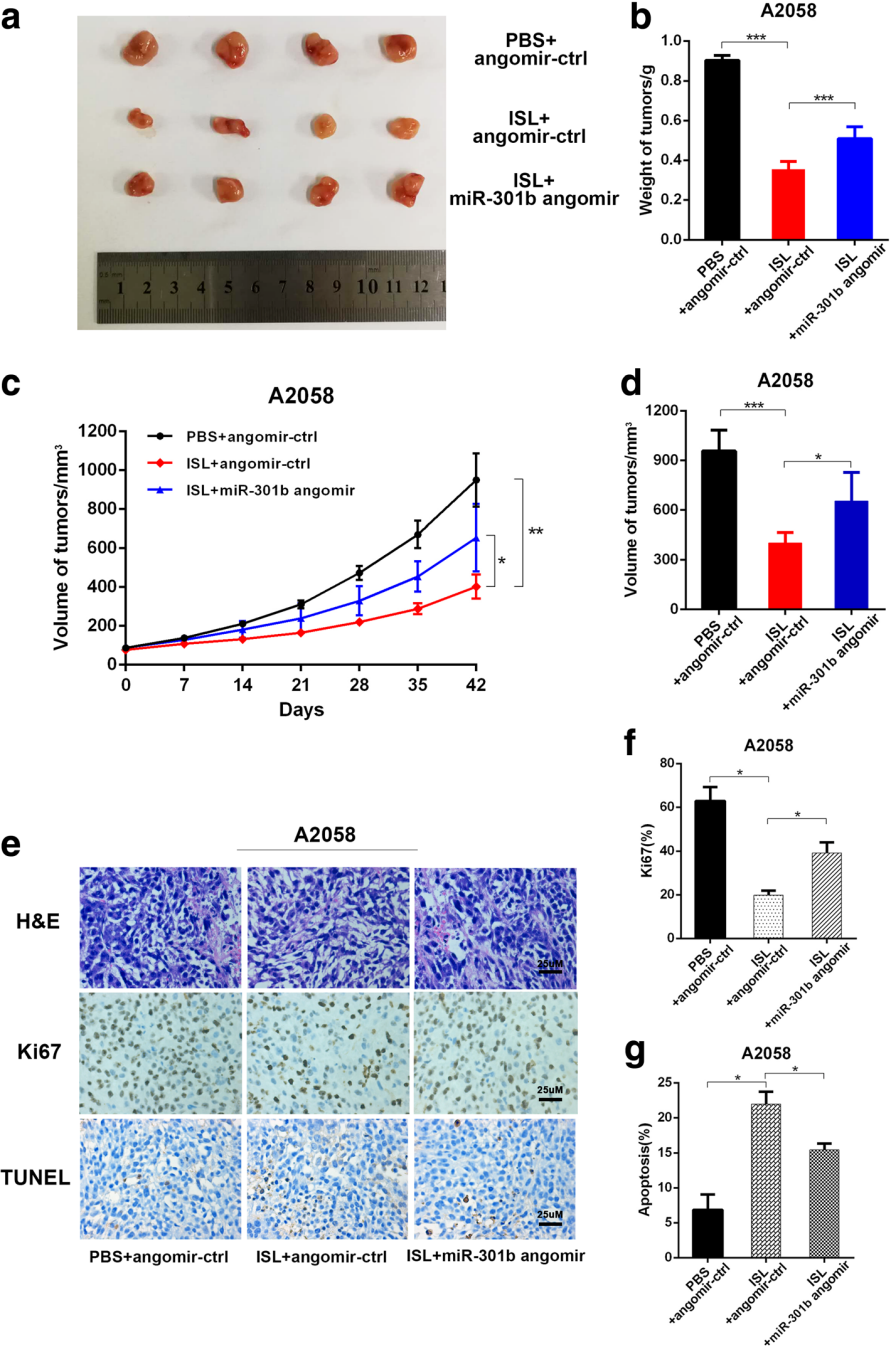
Silence of miR-301b enhances the anti-cancer effect of ISL on melanoma in vivo. A2058 cells were implanted subcutaneously into immunocompromised mice. When the tumors reached 80 mm^3^ in volume, the mice received intratumoral administration of miR-301b angomir or corresponding negative control and intraperitoneal administration of ISL every other day. The tumors were excised 42 days after treatment for analysis. **a** Representative images of tumor form in each group. **b**-**d** Quantification of volume, weight and growth curve of tumors in each group. **e** Representative images of H&E staining and immunohistochemical staining of Ki67 and TUNEL in tumor sections of each group. **f**-**g** Quantification of percentage of Ki67 and TUNEL positive cells in tumor sections. *P < 0.05, **P < 0.01, ***P < 0.001, n = 3

To validate the therapeutic efficacy of this treatment, a xenograft tumor mouse model was constructed after subcutaneous injection of 3 × 10^6^ A2058 cells into the right posterior flank of nude mice respectively. The tumor size was observed every seven days. As we observed in previous study, miR-301b played a counteractive role in the anti-cancer activity of ISL against melanoma, so when the tumor reached a discernible size of 80mm^3^, drugs were administrated every other day. 42 days after treatment, we excised the tumor from the sacrificed mouse and found the tumor size of ISL + angomir control group was much smaller than that in PBS + angomir control group, and this effect is slightly restored when the expression of miR-301b was up regulated(Fig. 5a, b, d). The same result was observed in the tumor growth curve drawn from the estimated tumor size (Fig. 5c). To further elucidate the under mechanism of anti-cancer effect on tumor mass, we performed histological analysis of tumor sections. TUNEL staining was performed to assess tumor apoptosis. As shown in Fig. 5e and g, administration of ISL + angomir control significantly induced cell apoptosis, indicated by higher percentage of TUNEL positive cell in the tumor, and this effect became more distinct when miR-301b angomir was administrated. Next, we stained the tumor sections with Ki67, a vital cellular marker for mitosis, and the result demonstrated that ISL alone was able to reduce the number of Ki67 positive cells dramatically, which was partially ablated by the presence of angomir miR-301b (Fig. 5e and f). These results strongly suggested that miR-301b exerted suppressive effect on the therapeutic activity against melanoma.

### MiR-301b regulates the inhibition of ISL on melanoma by targeting LRIG1

**Fig. 6:**
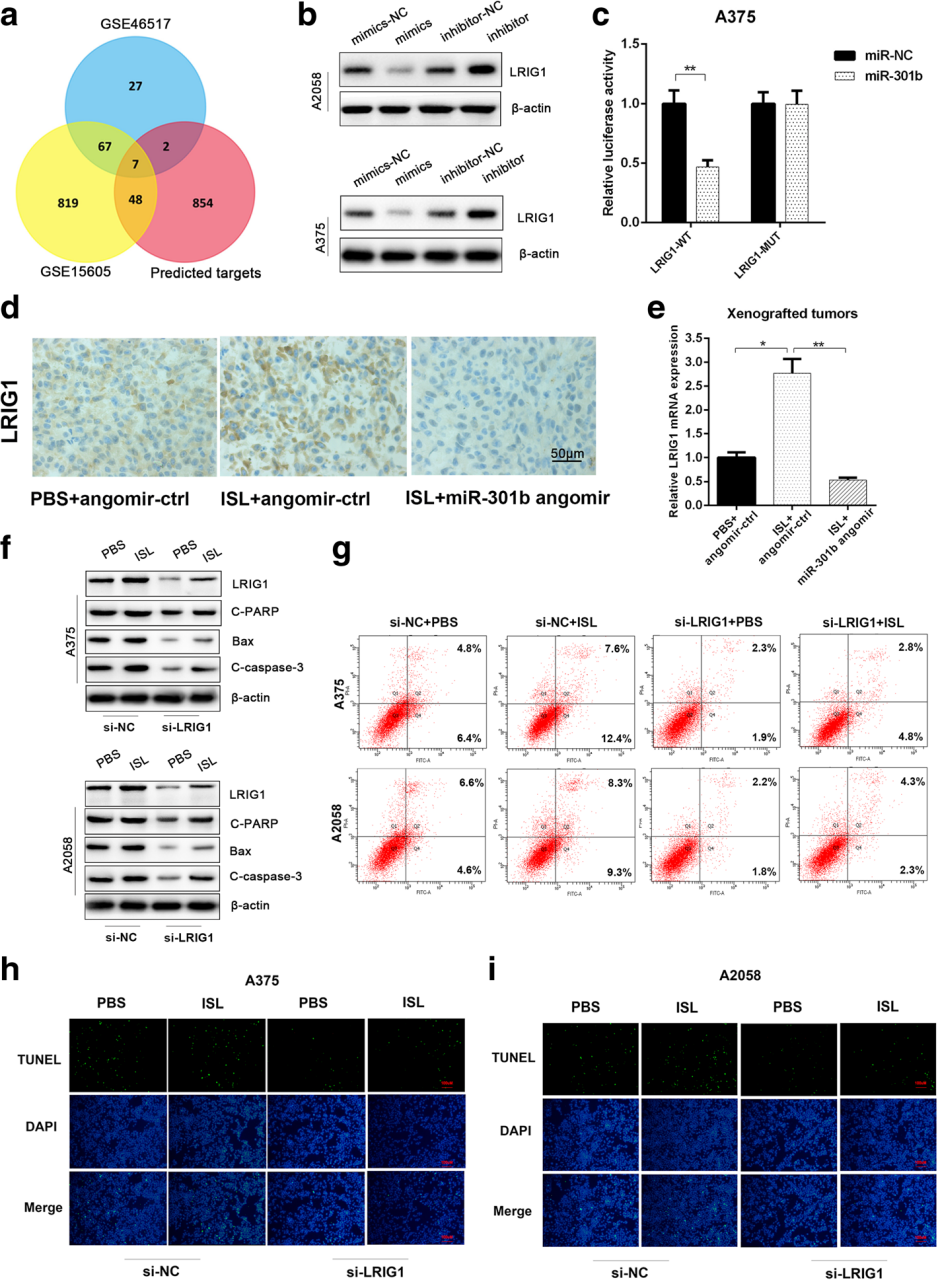
MiR-301b regulates the inhibitory effect of ISL on melanoma by functionally targeting LRIG1. **a** 7 common target genes predicted by TargetScan and GEO database were selected as potential target genes of miR-301b. **b** The western blot analysis of LRIG1 protein level in A375 and A2058 cells after being treated with miR-301b minics/inhibitor. **c** The effect of miR-NC, miR-301b on luciferase (luc) activity in A375 cells transfected with either the LRIG1–3’UTR WT or the LRIG1–3’UTR MUT. *P < 0.05 vs miR-NC group, *n* = 3. **d** Immunohistochemical analysis of LRIG1 in tumor sections of PBS + angomir-ctr group, ISL + angomir-ctr group and ISL + miR-301b angomir group. **e** The RT-qPCR analysis of LRIG1 in tumor samples of PBS + angomir-ctr group, ISL + angomir-ctr group and ISL+ miR-301b angomir group. **f** The western blot analysis of apoptosis associated proteins(LRIG1, c-PARP, Bax, bcl-2, C-caspase-3) in ISL treated A375 and A2058 cells which were transfected with LRIG1 siRNA or scramble vector in advance. **g** The flow cytometry analysis of apoptosis in ISL treated A375 and A2058 cells which were transfected with LRIG1 siRNA or scramble vector in advance. **h**, **i** Representative images of TUNEL staining in ISL treated A375 and A2058 cells which were transfected with LRIG1 siRNA or scramble vector in advance. *P < 0.05, **P < 0.01 vs PBS + angomir-ctr group, *n* = 3

To investigate the underlying mechanism of ISL mediated melanoma inhibition modulated by miR-301b, we used miRBase and GEO database to predict the target genes of miR-301b, and the common genes of two database was considered the potential target genes, the result indicated that 7 genes was tightly correlated with miR-301b (Fig. 6a). In order to identify the gene most closely related to miR-301b, we transfected A375 cells with miR-301b mimic or miR-301b inhibitor and measure the expression alteration of the 7 candidate target genes, as depicted in Additional file 4: Figure S3A, LRIG1 exhibited the most drastic expression change in the treatment. TargetScan displayed that LRIG1 has a miR-301b binding site in its 3’untranslated region (UTR) (Additional file 4: Figure S3B).

To test whether LRIG1 has a binding site for miR-301b, the effect of miR-301b mimic or miR-301b inhibitor on LRIG1 mRNA and protein expression levels in tumor cells were examined. As shown in Fig. 6b and Additional file 4: Figure S3D, the protein and mRNA level of LRIG1 were downregulated by miR-301b mimics and upregulated by miR-301b inhibitor. To investigate whether miR-301b targets LRIG1 directly, we constructed luciferase reporters containing a wild-type(WT) LRIG1 3’UTR or a mutant LRIG1 3’UTR sequence of the miR-301b binding site, and we found that miR-301b significantly suppressed the luciferase reporter activity of the WT miR-301b-3’UTR, but not in that of the mutant miR-301b-3’UTR (Fig. 6c).

To investigate whether miR-301b functionally targeted LRIG1 in the inhibition of melanoma, we firstly performed immunohistochemical assay to detect LRIG1 protein in the xenografted tumors. As depicted in Fig. 6d, ISL alone upregulated the protein level of LRIG1 dramatically, but the effect was obviously attenuated when miR-301b angomir was injected, and the consistent tendency was drawn in mRNA level of LRIG1(Fig. 6e). Next, we probed the role LRIG1 played in anti-cancer activity of ISL, we showed that ISL induced cell apoptosis was reversed when LRIG1 was knocked down, the expression level of pro-apoptotic genes(C-PARP, Bax, C-caspase-3) was enhanced (Fig. 6f, g and Additional file 4: Figure S3E, F, G). As revealed in Fig. 6h, i and Additional file 4: Figure S3G, H, the proportion of TUNEL positive cells caused by ISL treatment was reduced by the silence of LRIG1. Taken together, these results manifested that miR-301b mediates the anti-cancer effect of ISL on melanoma by functionally targeting LRIG1.

### Effect of si-LRIG1 on tumor growth in vivo

**Fig. 7:**
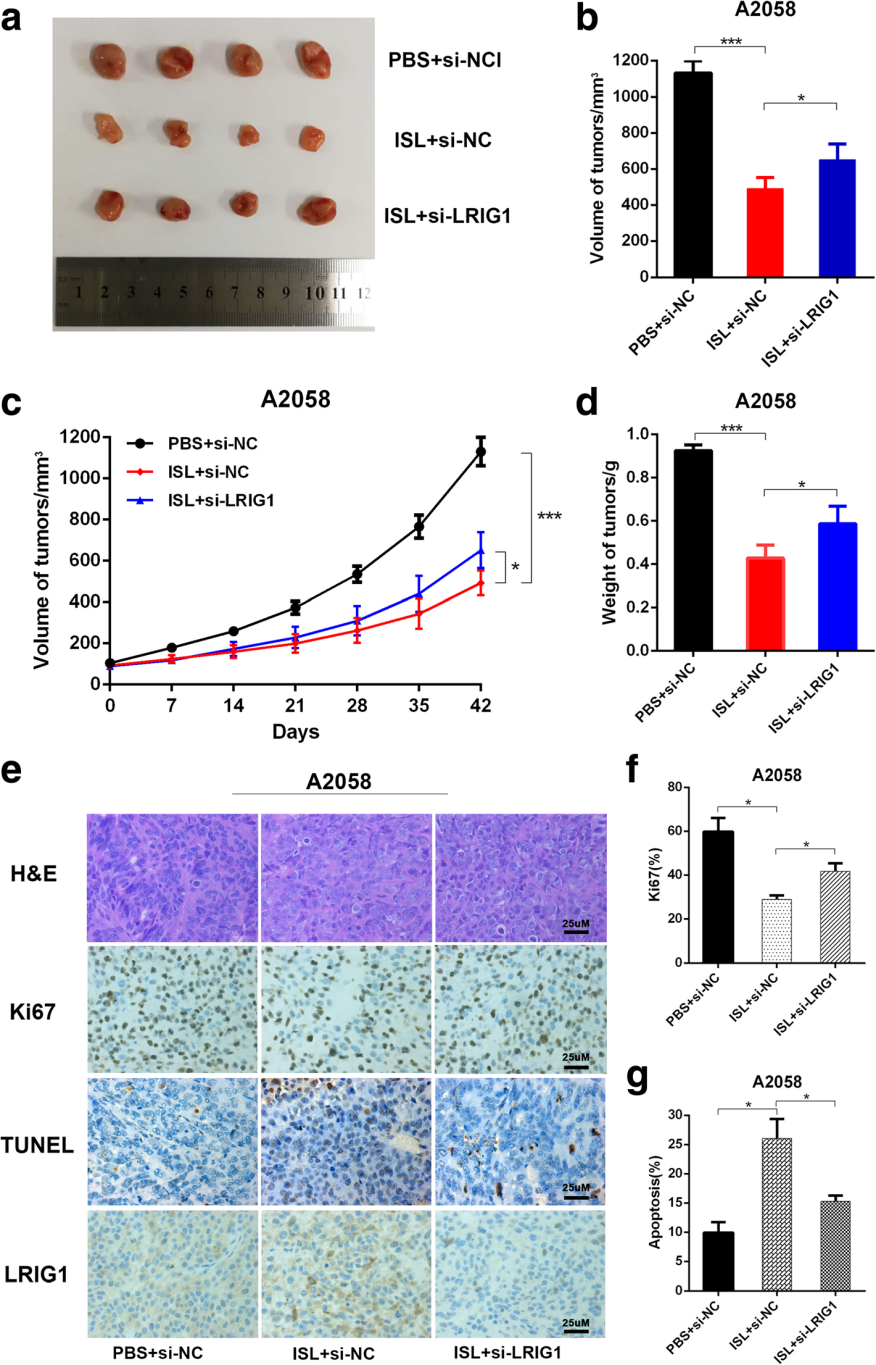
LRIG1 inhibits tumor growth in vivo. A2058 cells were implanted subcutaneously into immunocompromised mice. When the tumors reached 80 mm^3^ in volume, the mice received intratumoral administration of LRIG1 siRNA or scramble vector and intraperitoneal administration of ISL every other day. The tumors were harvested 42 days after treatment for analysis. **a** Representative images of tumor form in each group. **b**-**d** Quantification of volume, weight and growth curve of tumors in each group. **e** Representative images of H&E staining and immunohistochemical staining of Ki67 and TUNEL in tumor sections of each group. **f**-**g** Quantification of percentage of Ki67 and TUNEL positive cells in tumor sections. *P < 0.05, ***P < 0.001, n = 3

To validate the therapeutic effect of si-LRIG1 on melanoma, we constructed the xenografted tumor mouse model as described previously. When the tumor reached a desired size of 50 mm^3^, we intratumorally injected si-LRIG1 control/si-LRIG1 and intraperitoneally injected ISL/PBS. During the treatment, the size of tumors was monitored. 42 days after treatment, tumors were harvested and the volume and weight were measured. As shown in Fig. 7a, b and d, the average volume and weight of tumors were significantly reduced by ISL treatment, and the effect was abated dramatically by si-LRIG1. And tumor growth curve indicated that tumor growth rate was obviously suppressed by ISL, and the activity was reversed when LRIG1 was knocked down (Fig. 7c).

To test the impact of si-LRIG1 on tumor cell apoptosis and proliferation, we stained the tumor sections with H&E, TUNEL and Ki67. As depicted in Fig. 7e, f, g, ISL triggered cell apoptosis was significantly mitigated by si-LRIG1, accordingly, cell mitosis inhibition induced by ISL was alleviated when LRIG1 was knocked down. The results above revealed that the therapeutic effect of ISL on melanoma was impaired when the expression of LRIG1 was downregulated.

### Clinical correlation between LRIG1 and miR-301b

**Fig. 8:**
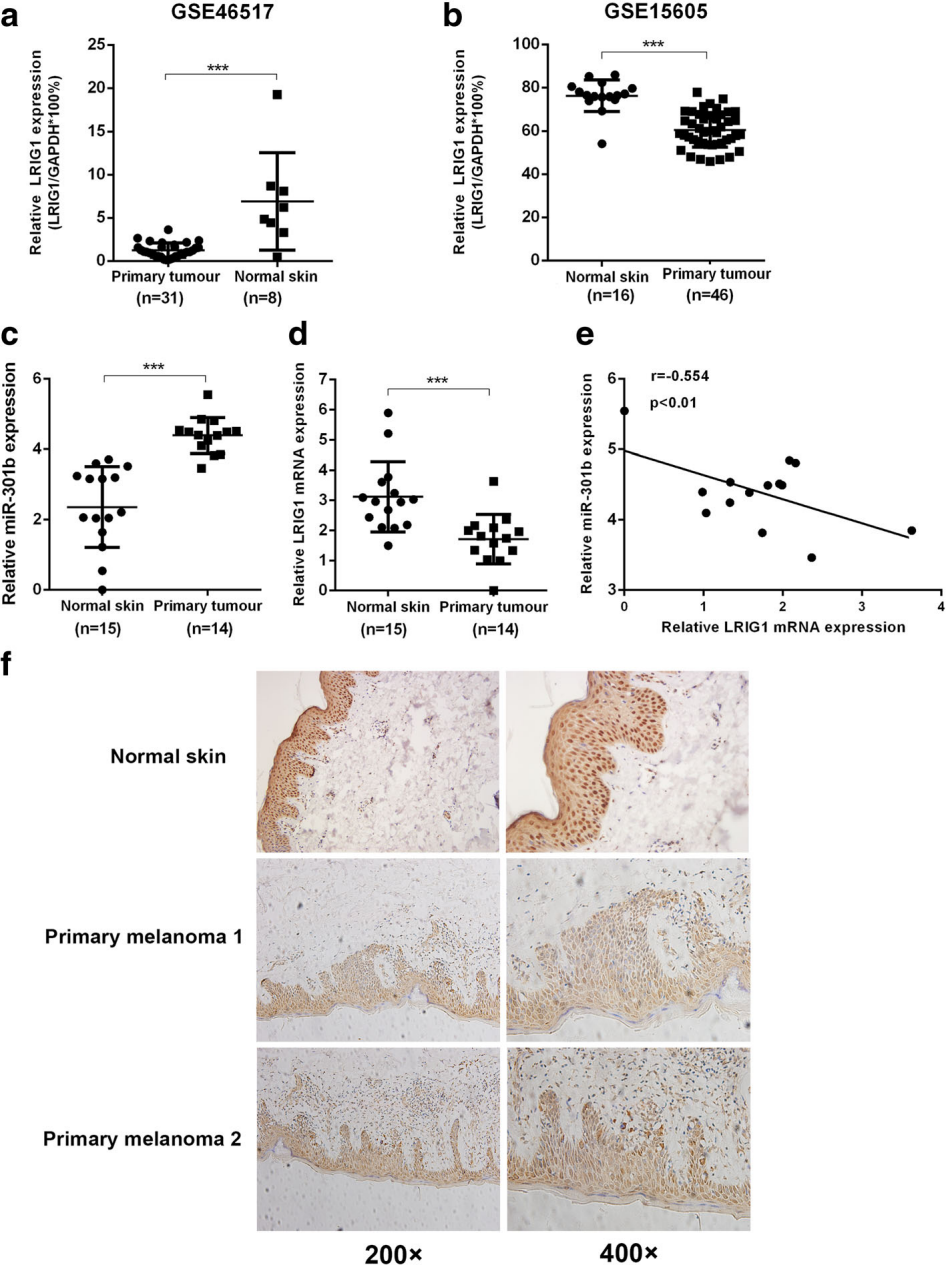
LRIG1 and miR-301b are negatively correlated clinically. **a**, **b** Expression of LRIG1 in primary melanoma sample and normal skin determined by GSE46517 and GSE15605 dataset from GEO database (calculated as the ratio of LRIG1 and GAPDH). **c**, **d** RT-qPCR analysis of the mRNA level of LRIG1 and miR-301b in primary melanoma samples and normal skin samples. **e** Correlation of LRIG1 and miR-301b in primary melanoma samples and normal skin samples. **f** Representative images of immunohistochemical of LRIG1 in primary melanoma samples and normal skin samples. ***P < 0.001

To gain further insight into the correlation between LRIG1 and miR-301b clinically, we used dataset GSE46517 and GSE15606 from GEO database to measure the expression level of LRIG1 in primary tumor tissue and normal skin tissue. As shown in Fig. 8a and b, relative expression of LRIG1 was higher in primary tissue compared to normal skin tissue. Next, we examined the mRNA level of LRIG1 and miR-301b in tumor and normal skin samples, and the results showed that mRNA level of LRIG1 was lower and mRNA level of miR-301b higher in tumor tissue compared to that of in normal skin tissue (Fig. 8c and d). And it demonstrated that the correlation between relative miR-301b expression and relative LRIG1 expression was negative significant (Fig. 8e). Immunohistochemical staining showed that protein level in melanoma tissue was lower in comparison to normal skin tissue. These results revealed that the expression of miR-301b is negatively correlated with that of LRIG1 in melanoma.

## Discussion

Isoliquiritigenin (ISL) is the active medicinal component isolated from the root of licorice, which possesses great therapeutic value in the treatment of various human diseases based on its biological properties such as anti-oxidant, anti-inflammation and anti-cancer activities. In particular, the anti-cancer effect of ISL has been researched in wide-spectrum of malignant tumors. For instance, the cycle of prostate cancer cells was arrested and the apoptosis was increased after ISL application [37]. Application of ISL also showed desirable efficacy on the treatment of human brain glioma by stimulating the differentiation of glioma stem cells [38]. And ISL was deemed to enhance WIF1 gene expression via promoting the demethylation of its promoter, which exerted the anti-cancer activity against breast cancer [39]. And an alternative study has proposed that ISL inhibited breast cancer metastasis by downregulating COX-2 and CYP4A signaling [40]. In human lung cancer cells, activation of wild type or mutant EGFR was suggested to mediate the ISL induced apoptosis, p53 and p21 upregulation was also verified to be part of the mechanism [41, 42]. In malignant melanoma, ISL induced reprogramming of melanoma cells by activating mTORC2-AKT-GSK3β signaling,and it has the same effect on mouse melanoma cells [30, 31]. In accordance with these findings, we showed that ISL significantly suppressed the cell growth and induced apoptosis of two human melanoma cell lines. Our data confirmed the inhibitory role of ISL played in the treatment of melanoma. We used high-throughput sequencing to screen out the most differentially expressed miRNAs in response to the ISL treatment, and miR-301b demonstrated the most dramatic expression alteration.

Cumulative researches of the functions of miR-301b in tumor progression have been carried out in the past few years. MiR-301b has been shown to promote the proliferation, migration and aggressiveness of human bladder cancer cells by targeting EGR1 or through FAK and Akt phosphorylation by regulating PTEN [43, 44]. The underlying mechanism of the malignant pancreatic cancer aggressiveness driven by migration inhibitory factor was through upregulation of miR-301b [45]. And the diagnostic indicative value of miR-301b has also been extensively interrogated, miR-301b along with other miRNAs were abundant in plasma of patients diagnosed with Acute myelocytic leukemia(AML), revealing that it may be a sensitive therapeutic signature of AML. Consistent with these studies, we provided proofs in favor of the crucial role of miR-301b in mediating the anti-cancer effect of ISL on melanoma. Relative expression of miR-301b in A375 and A2058 cells was significantly restrained by the ISL administration, and ectopic introduction of miR-301b partially abolished the ISL elicited suppression on melanoma cells. Intratumoral overexpression of miR-301b ablated the growth inhibition and apoptosis promotion induced by ISL in vivo.

Mechanistically, we traced the pathological role of miR-301b through identification of the candidate target genes. Based on GEO and bioinformatics analysis, we confirmed that LRIG1 exhibited the most significant expression change upon the treatment with miR-301b. LRIG1 belongs to LRIG gene family and functions as a tumor suppressor, and its high expression is associated with an increased survival in various tumor types including breast cancer, ovarian cancer, uterine cervical cancer, cutaneous squamous cell carcinoma, nasopharyngeal, oropharyngeal cancer, non-small cell lung cancer and hepatocellular carcinoma [46, 47]. Our data demonstrated that LRIG1 is a potential modulator in anti-cancer activity of ISL against melanoma, as specific knockdown of LRIG1 decreased cell apoptosis exerted by ISL. Invalidation of the relationship between LRIG1 and miR-301b suggested that the expression of LRIG1 in melanoma cells declined drastically upon the ectopic expression of miR-301b, and intratumoral expression level of LRIG1 was significantly restored when miR-301b was silenced. At the genetic level, we found that miR-301b had a binding site on the 3’UTR of LRIG1. Clinically, the expression of LRIG1 in tumor samples obtained from melanoma patients was decreased compared to normal skin samples, while the expression of miR-301b showed the contrary tendency, indicating a negative correlation between LRIG1 and miR-301b. This experimental evidence denoted that LRIG1 is a functional target of miR-301b and mediates the biological behavior of ISL in the treatment of melanoma.

## Conclusion

In summary, our research firstly demonstrated that the inhibitory effect of ISL exerted on melanoma proliferation and apoptosis was mediated by miR-301b, underlying which LRIG1 is the potential target of this process. This may provide a novel insight for the treatment of melanoma in the future.

## Additional files

Additional file 1: **Figure S2.** (A, B)RT-qPCR analysis of the mRNA level of miR301b in ISL treated A375 and A2058 cells which were transfected with miR301b or control(NC). *P < 0.05, **P < 0.01 vs PBS Treated group. (C, D)Western blot analysis of the protein level of apoptosis associated proteins(c-PARP, Bax, bcl-2, cleaved-caspase-3) in ISL treated A375 and A2058 cells which were transfected with miR301b or miR-NC. *P < 0.05, **P < 0.01 vs miR-NC Treated in PBS groups. ^#^*P* < 0.05, ^##^*P* < 0.01 vs miR-NC Treated in ISL groups. (TIF 13582 kb)

Additional file 2: **Table S1.** Sequences of mRNA PCR primers used in this study. (DOCX 19 kb)

Additional file 3: **Figure S1.** ISL inhibits cell proliferation and induces cell apoptosis in melanoma cells in vitro. (A) MEWO cells were treated with ISL (0, 10, 20, 40, 80 μM) for 24 h, and cell viability was analyzed by CCK-8 assay. (B) MEWO cells were treated with 20 μM ISL, cell proliferation at indicated time (24, 48, 72 h) was measured by CCK-8 assay. (C, D) Flow cytometry analysis of apoptosis of MEWO cells after being treated with ISL (0, 10, 20 μM) for 24 h. (E, F) Representative images and quantification of colony formation of MEWO cells after being treated with ISL (0, 5, 10 Μm). (G, H)Western blot analysis of the protein level of apoptosis associated proteins(bcl-2, bax, parp, cleaved-caspase-3) in ISL treated A375 and A2058 cells. *P < 0.05, **P < 0.01, ***P < 0.001 vs ISL(0 μM) treated group. *n* = 3. (TIF 25527 kb)

Additional file 4: **Figure S3.** (A)RT-qPCR analysis of the mRNA level alteration of 7 common target genes of miR301b in A375 and A2058 cells after being transfected with miR301b mimic/NC or treated with miR301b inhibitor/NC. *P < 0.05 vs NC. (B)Design of luciferase reporters with the WT Akt3 3’UTR (Akt3–3’UTR WT) or the site-directed mutant Akt3 3’UTR (Akt3–3’UTR MUT). (C)RT-qPCR analysis of miR301b level in A375 and A2058 cells after being transfected with miR301b mimic/NC or treated with miR301b inhibitor/NC. **P < 0.01 vs NC. (D)RT-qPCR analysis of the mRNA level of LRIG1 in A375 and A2058 cells after being transfected with miR301b mimic/NC or treated with miR301b inhibitor/NC. **P < 0.01 vs NC.*P < 0.05 vs NC. (E, F)Western blot analysis of the protein level of apoptosis associated proteins(LRIG1, c-PARP, Bax, cleaved-caspase-3) in ISL treated A375 and A2058 cells which were transfected with si-LRIG1 or control(NC). *P < 0.05, **P < 0.01 vs PBS Treated in si-NC groups. ^#^*P* < 0.05, ^##^*P* < 0.01 vs PBS Treated in si-LRIG1 groups. (G)Flow cytometry analysis of cell apoptosis in ISL treated A375 and A2058 cells which were transfected with si-LRIG1 or si-NC.*P < 0.05, **P < 0.01 vs PBS + si-NC. (H)Quantification of TUNEL positive cells in ISL treated A375 and A2058 cells which were transfected with si-LRIG1 or si-NC.*P < 0.05, **P < 0.01 vs PBS + si-NC. (TIF 25520 kb)

## Abbreviations

ISL: Isoliquiritigenin
LRIG1: leucine rich repeats and immunoglobulin like domains 1
PARP: poly ADP-ribose polymerase
siRNA: small interfering RNA
