# Supplementary figures and images for: Isoliquiritigenin suppresses human melanoma growth by targeting miR-301b/LRIG1 signaling
Source: J Exp Clin Cancer Res. 2018 Aug 6;37:184. doi: 10.1186/s13046-018-0844-x (PMC6091185; doi:10.1186/s13046-018-0844-x)

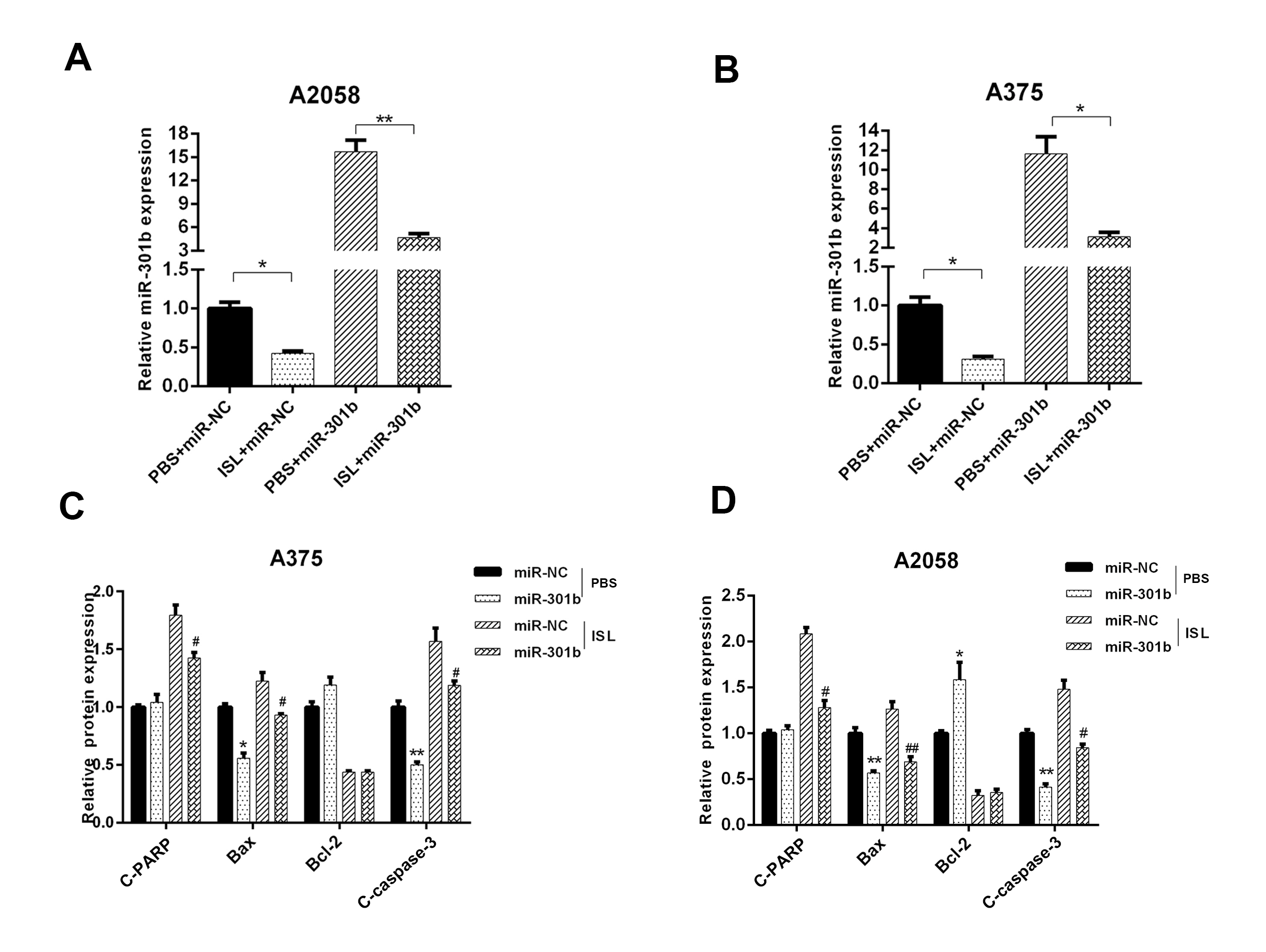

Supplement: Supplementary file 1 — Figure S2. (A, B)RT-qPCR analysis of the mRNA level of miR301b in ISL treated A375 and A2058 cells which were transfected with miR301b or control(NC). *P < 0.05, **P < 0.01 vs PBS Treated group. (C, D)Western blot analysis of the protein level of apoptosis associated proteins(c-PARP, Bax, bcl-2, cleaved-caspase-3) in ISL treated A375 and A2058 cells which were transfected with miR301b or miR-NC. *P < 0.05, **P < 0.01 vs miR-NC Treated in PBS groups. #P < 0.05, ##P < 0.01 vs miR-NC Treated in ISL groups. (TIF 13582 kb) [file 13046_2018_844_MOESM1_ESM.tif]

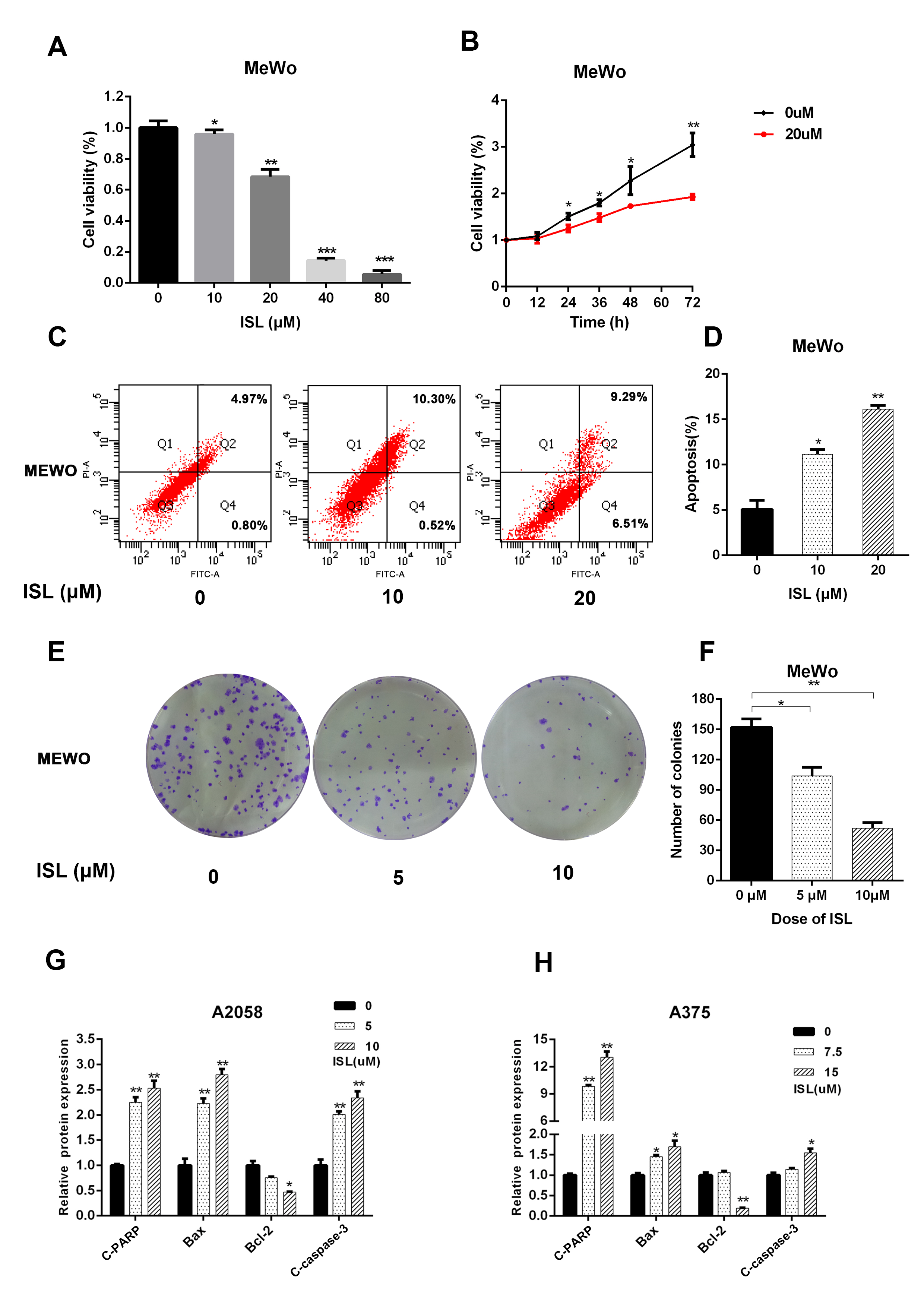

Supplement: Supplementary file 3 — Figure S1. ISL inhibits cell proliferation and induces cell apoptosis in melanoma cells in vitro. (A) MEWO cells were treated with ISL (0, 10, 20, 40, 80 μM) for 24 h, and cell viability was analyzed by CCK-8 assay. (B) MEWO cells were treated with 20 μM ISL, cell proliferation at indicated time (24, 48, 72 h) was measured by CCK-8 assay. (C, D) Flow cytometry analysis of apoptosis of MEWO cells after being treated with ISL (0, 10, 20 μM) for 24 h. (E, F) Representative images and quantification of colony formation of MEWO cells after being treated with ISL (0, 5, 10 Μm). (G, H)Western blot analysis of the protein level of apoptosis associated proteins(bcl-2, bax, parp, cleaved-caspase-3) in ISL treated A375 and A2058 cells. *P < 0.05, **P < 0.01, ***P < 0.001 vs ISL(0 μM) treated group. n = 3. (TIF 25527 kb) [file 13046_2018_844_MOESM3_ESM.tif]

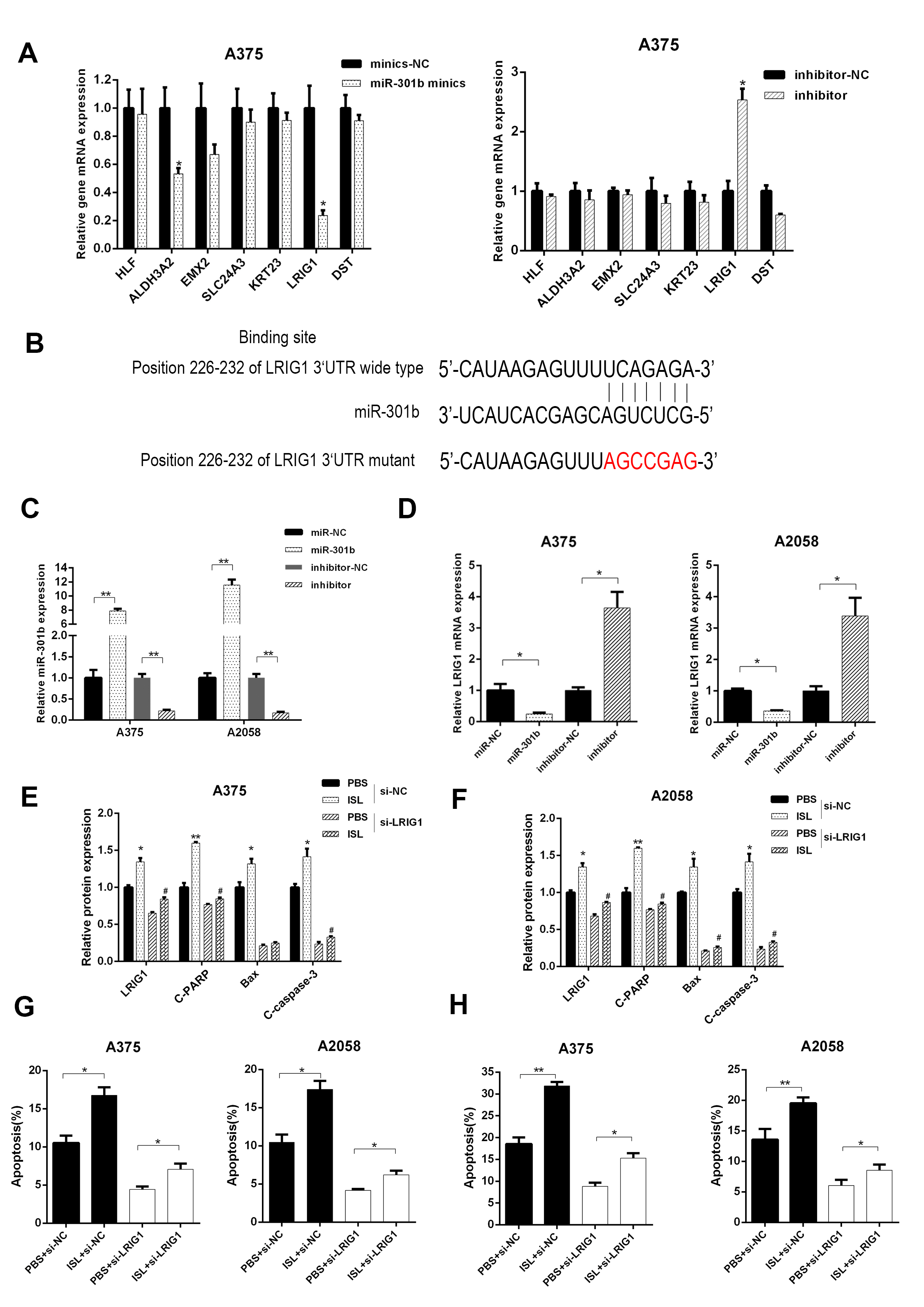

Supplement: Supplementary file 4 — Figure S3. (A)RT-qPCR analysis of the mRNA level alteration of 7 common target genes of miR301b in A375 and A2058 cells after being transfected with miR301b mimic/NC or treated with miR301b inhibitor/NC. *P < 0.05 vs NC. (B)Design of luciferase reporters with the WT Akt3 3’UTR (Akt3–3’UTR WT) or the site-directed mutant Akt3 3’UTR (Akt3–3’UTR MUT). (C)RT-qPCR analysis of miR301b level in A375 and A2058 cells after being transfected with miR301b mimic/NC or treated with miR301b inhibitor/NC. **P < 0.01 vs NC. (D)RT-qPCR analysis of the mRNA level of LRIG1 in A375 and A2058 cells after being transfected with miR301b mimic/NC or treated with miR301b inhibitor/NC. **P < 0.01 vs NC.*P < 0.05 vs NC. (E, F)Western blot analysis of the protein level of apoptosis associated proteins(LRIG1, c-PARP, Bax, cleaved-caspase-3) in ISL treated A375 and A2058 cells which were transfected with si-LRIG1 or control(NC). *P < 0.05, **P < 0.01 vs PBS Treated in si-NC groups. #P < 0.05, ##P < 0.01 vs PBS Treated in si-LRIG1 groups. (G)Flow cytometry analysis of cell apoptosis in ISL treated A375 and A2058 cells which were transfected with si-LRIG1 or si-NC.*P < 0.05, **P < 0.01 vs PBS + si-NC. (H)Quantification of TUNEL positive cells in ISL treated A375 and A2058 cells which were transfected with si-LRIG1 or si-NC.*P < 0.05, **P < 0.01 vs PBS + si-NC. (TIF 25520 kb) [file 13046_2018_844_MOESM4_ESM.tif]
