# Supplementary material for: Isoliquiritigenin suppresses human melanoma growth by targeting miR-301b/LRIG1 signaling
Source: J Exp Clin Cancer Res. 2018 Aug 6;37:184. doi: 10.1186/s13046-018-0844-x (PMC6091185; doi:10.1186/s13046-018-0844-x)
Supplement: Supplementary file 2 — Table S1. Sequences of mRNA PCR primers used in this study. (DOCX 19 kb) [file 13046_2018_844_MOESM2_ESM.docx]

**Table S1 Sequences of mRNA PCR primers used in this study.**

| HLF | Forward(5’-3’) | GCCAGCAAACCGCAATAC |
| --- | --- | --- |
|  | Reverse(5’-3’) | CCTCAAGTCAGCCACCTCC |
| ALDH3A2 | Forward(5’-3’) | TGCCAAGCATCTGACCCC |
|  | Reverse(5’-3’) | CCTTCAAGCAAACTTAGTATCCTCT |
| EMX2 | Forward(5’-3’) | CGCTAACTCCAGCCCCATA |
|  | Reverse(5’-3’) | GCCTCGGCGAACACCA |
| KRT23 | Forward(5’-3’) | CAGAGCAGACAAGGTGACATCC |
|  | Reverse(5’-3’) | GCGTCAGTTCCTCCTCATAGTG |
| LRIG1 | Forward(5’-3’) | ATTGACCCTGCTGGTTTTGA |
|  | Reverse(5’-3’) | GGTGTTCCGCACTTCCGT |
| DST | Forward(5’-3’) | GCCCTCGTAAAACTCTATGAAAC |
|  | Reverse(5’-3’) | GCCCTCTAAGTCCCGTAACCT |
| β-actin | Forward(5’-3’) | AATCGTGCGTGACATTAAGGAG |
|  | Reverse(5’-3’) | ACGTGTTGGCGTAACAGGTCTT |
| SLC24A3- | Forward(5’-3’) | GCTGCTCTGGTCGCTGTCG |
|  | Reverse(5’-3’) | CCCGCTGCCATGAATGTG |
